# The gut mucin-microbiota interactions: a missing key to optimizing endurance performance

**DOI:** 10.3389/fphys.2023.1284423

**Published:** 2023-11-22

**Authors:** Allison Clark, Núria Mach

**Affiliations:** ^1^ Universitat Oberta de Catalunya, Universitat de Catalunya, Barcelona, Spain; ^2^ Interactions hôtes-agents pathogènes, Université de Toulouse, Institut national de recherche pour l’agriculture, l’alimentation et l’environnement, École nationale vétérinaire de Toulouse, Toulouse, France

**Keywords:** athletes, endurance, glycome, dietary glycans, microbiota, mucins, performance

## Abstract

Endurance athletes offer unique physiology and metabolism compared to sedentary individuals. Athletes training at high intensities for prolonged periods are at risk for gastrointestinal disturbances. An important factor in endurance performance is the integrity and function of the gut barrier, which primarily depends on heavily *O*-glycosylated mucins. Emerging evidence shows a complex bidirectional dialogue between glycans on mucins and gut microorganisms. This review emphasizes the importance of the crosstalk between the gut microbiome and host mucus mucins and some of the mechanisms underlying this symbiosis. The contribution of mucin glycans to the composition and functionality of the gut microbiome is discussed, as well as the persuasive impact of the gut microbiome on mucin composition, thickness, and immune and metabolic functions. Lastly, we propose natural and synthetic glycans supplements to improve intestinal mucus production and barrier function, offering new opportunities to enhance endurance athletes’ performance and gut health.

## Introduction

Endurance exercise involves prolonged cardiovascular efforts—such as running, cross-country skiing, cycling, aerobic exercise, or swimming (Joyner and Coyle, 2008; Clark and Mach, 2016; Mach and Fuster-Botella, 2017). Endurance exercise performance primarily depends on physiological adaptations to sustain the metabolic and thermoregulatory demands of such activity (Shave et al., 2017), including coordinated muscle contractions, fatty acid oxidation, increased use of glycogen stores, mitochondrial biogenesis, increased reactive oxygen species (ROS) production, electrolyte rebalance (De Oliveira et al., 2014), and intestinal mucosa adaptation (Koehler et al., 2021).

An estimated 30%–50% of endurance athletes suffer from acute gastrointestinal complaints, including diarrhea, vomiting, nausea, and ischemia (de Oliveira and Burini, 2014), which can cause temporary gut microbial imbalances, intestinal barrier damage, and increased gut permeability and inflammation (Koehler et al., 2021). Intestinal ischemic damage is caused as blood flows away from the gastrointestinal tract during intense exercise. Although blood flow is restored to the gut after exercise, long-lasting conditions might prevail (Waterman and Kapur, 2012). The risk of these digestive troubles is amplified when athletes push beyond their expected physical limits or when other immune and metabolic stressors are present, including altitude, elevated environmental temperature, fluid restriction, extreme trainability, exposure to novel pathogens during travel, lack of sleep, severe mental stress, malnutrition, or weight loss (Nieman, 2003; Neufer et al., 2015; Koehler et al., 2021).

Undoubtedly, intestinal health and homeostasis entail complex multifactorial processes whose mechanisms are still not fully understood. New evidence has shown that the gut microbiota, defined as the complex community of microorganisms that reside in the gut (Fischbach, 2018), impacts the gastrointestinal tract’s health (Fan and Pedersen, 2021). The human gut microbiota contains over 100 trillion microorganisms and up to 10 million non-redundant genes (Forster et al., 2016), with an enormous metabolic capacity. It spans over 2,000 bacterial species (Donaldson et al., 2015; Jousset et al., 2017) and various archaea, eukaryotic taxa, and viruses, with a density of ∼40–300 billion microorganisms in the colon (Vandeputte et al., 2016). Their functions include digestion and nutrient uptake, immune system regulation, metabolism, energy harvest, protection against pathogens, synthesis of vitamin and bioactive compounds, and brain function regulation (Rideout et al., 2014).

Each person has a relatively distinct but stable gut microbial community during adulthood (Lozupone et al., 2012). Diet and cultural factors are the main determinants of the gut ecosystem’s diversity and stability (Lozupone et al., 2012). But, antibiotics, anthelmintic use, stress, smoking, age, type of birth, pathophysiological conditions, and genetics can also drive microbial composition and function (Diener et al., 2022). Likewise, compelling evidence shows that exercise alters the function and structure of the gut ecosystem (Scheiman et al., 2019; Mohr et al., 2020; Barton et al., 2021; Morita et al., 2023). Endurance exercise has been studied as a significant long-term modifier of the intestinal microbiome composition and function in animal and human studies (Clarke et al., 2014; Barton et al., 2018; 2021; Scheiman et al., 2019; Plancade et al., 2019; Mach et al., 2021; Mach et al., 2022). Gut microbiota analyses have reported that human athletes had different microbiome compositions (defined by elevated abundances of Veillonellaceae, *Bacteroides*, *Prevotella*, *Methanobrevibacter,* or *Akkermansia* (Clarke et al., 2014; Petersen et al., 2017) with a high number of genes involved in carbohydrate and amino acid metabolism and short-chain fatty acids (SCFA) production (Barton et al., 2018).

**FIGURE 1:**
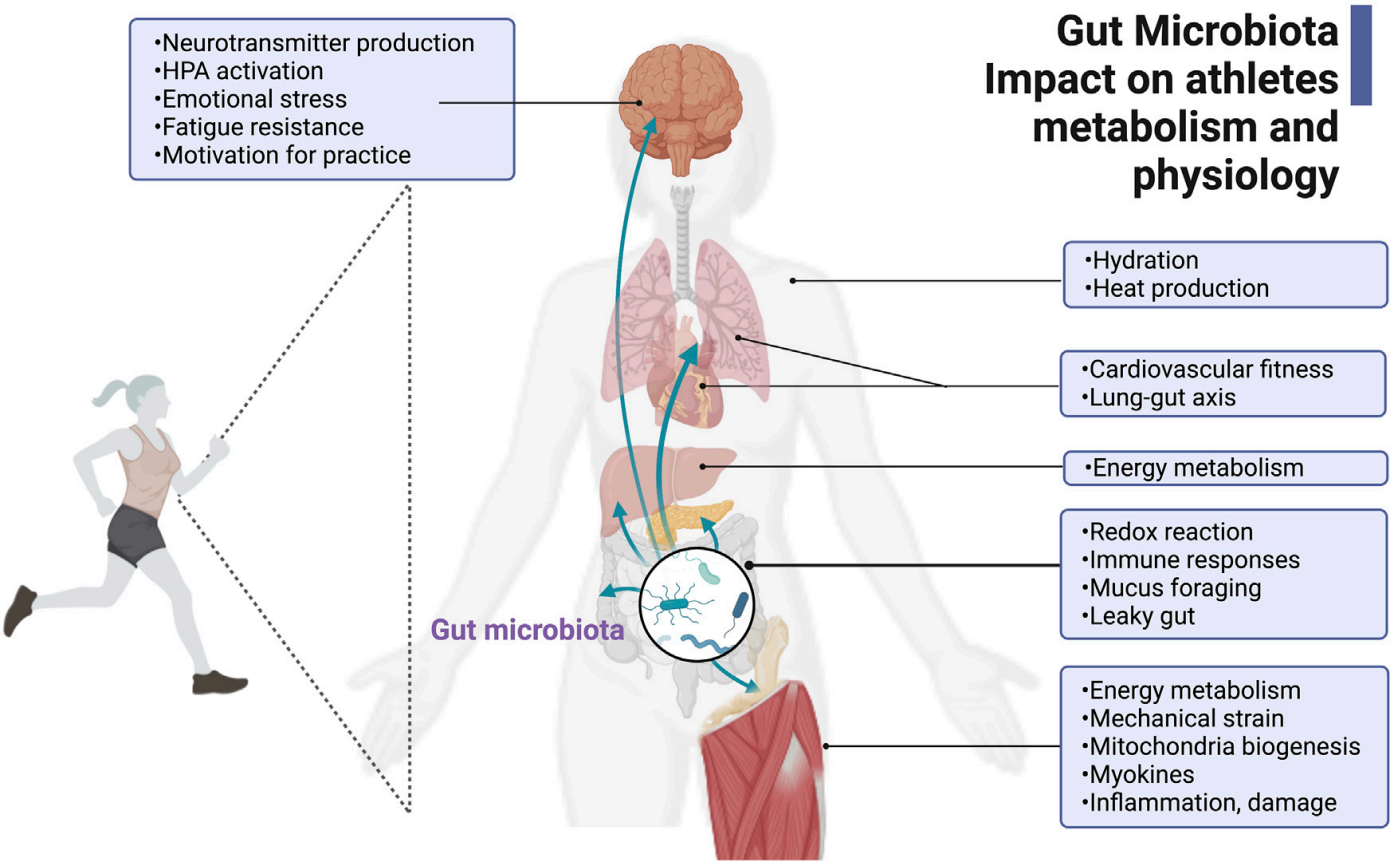
Gut microbiota’s impact on athlete’s metabolism and physiology. The gut microbiota in athletes is critical for its positive contributions to host energy metabolism and physiology during exercise, such as hydration, heat production, cardiovascular fitness, gut-lung axis regulation, redox reactions, immune responses, mucus foraging and intestinal mucosa protection, and homeostasis. The gut microbiota and its metabolites also regulate skeletal muscle function and neurotransmitter production related to fatigue, sensation, and motivation. This figure has been created with BioRender.com.

The growing interest in the gut microbiome in athletic performance has started to delineate potential ergogenic effects of the gut microbiome directly and indirectly through cytokines and metabolites that trigger the immune function and the gut-brain and gut-muscle axes (Estaki et al., 2016; Barton et al., 2018; Petersen et al., 2017; Durk et al., 2019; Scheiman et al., 2019; Ticinesi et al., 2019; Dohnalová et al., 2022). Figure 1 recent cause-and-effect studies in athletes’ microbiomes revealed that *Veillonella atypica* (Scheiman et al., 2019; Lundberg et al., 2021) or *Bacteroides uniformis* alone (Morita et al., 2023) are required to enhance athletic outputs. Scheiman et al. (2019) pointed to the lactate metabolism of *Veillonella atypica* as a potential basis for any ergogenic effect observed (Grosicki et al., 2019; Scheiman et al., 2019). Additionally, Dohnalová et al. (2022) proved, in a murine model, the influence of microbial production of endocannabinoid metabolites on motivation and mental states during exercise, potentially leading to improved performance.

Emerging evidence suggests that the gut microbial ecosystem also affects athletic performance by influencing the overall gut barrier function, including mucus layer composition and thickness, intestinal epithelial tight junction protein structure, antimicrobial peptide secretion, and goblet cells abundance (Pedersen, 2011; Leuchtmann et al., 2022). In the past, the intestinal mucus was mainly considered a simple lubricant for facilitating the progression of the food bolus and the stools in the gut (Paone and Cani, 2020). Today, it is known that the gut mucus layer is a crucial component of the intestinal barrier and plays a key role in maintaining gut health and homeostasis (Bansil and Turner, 2018; Nason et al., 2021). This mucus mainly comprises mucins, a family of high-molecular-weight glycoproteins with numerous *O*-linked glycans (also called carbohydrates, saccharides, or sugars) that confer unique physical and functional properties. Glycans from mucins are synthesized and secreted by goblet cells of the small intestine and colon or the surface mucous cells of the stomach (Cone, 2009). They are involved in several critical gastrointestinal functions. For example, the glycans on mucins provide a physical barrier that traps and clears particles and also serves as a chemical barrier that neutralizes toxins, allergens, pollutants, and pathogens, limiting the growth and colonization of pathogens and their adhesion and invasion of the intestinal epithelium (Schnaar, 2015; Goto et al., 2016; Bergstrom et al., 2017; Reily et al., 2019; Qin and Mahal, 2021; Brazil and Parkos, 2022). Mucin glycans also act as ligands for pathogen binding and receptors on immune cells. Thus, in addition to their role as a physical and chemical barrier, glycans on intestinal mucins have also been shown to have immune-modulating properties and may regulate inflammation in the gut (Schnaar, 2015; Reily et al., 2019; Qin and Mahal, 2021; Brazil and Parkos, 2022).

Notably, mucin glycans establish a symbiotic relationship with the microbiota (Glowacki and Martens, 2021; La Rosa et al., 2022). Mucins serve as an environmental niche and a food source for gut microorganisms (Koropatkin et al., 2012; Belzer, 2022). While most gut bacteria use dietary fibers and starches as a nutrient source, genera such as *Bacteroides, Bifidobacterium, Ruminococcus,* and *Akkermansia* encode thousands of carbohydrate-active enzymes (CAZymes) and associated transport systems to depolymerize and ferment glycans on mucins into CO_2_, H_2_, and SCFAs (Crouch et al., 2020; Taleb et al., 2022). As a result, these metabolites can have, in turn, local effects in the gut, including modifications of production and quality of mucins, regulation of tight junction proteins and immune system, or be absorbed into the bloodstream and affect systemic functions (Koropatkin et al., 2012). There is a clear need for further investigation to understand the functioning of the mucus barrier and gut health, especially during endurance exercise. Indeed, a lack of mucus production or a change in the properties and composition of mucus glycans can result in dry or thick mucus, which leads to unshielded and dehydrated intestinal epithelial surfaces that are prone to infection, wounding, permeability, and risk of endotoxemia (Werlang et al., 2019).

Despite an increasing appreciation of the importance of gut mucin composition and structure for gut health (van Putten and Strijbis, 2017; Taleb et al., 2022), their possible role in improving athletic performance has yet to be assessed. In addition to affecting the gut ecosystem, physical activity may also directly and indirectly impact mucin production in the gut. The relationship between physical activity, the gut microbiota, and mucins, coupled with the considerable malleability of the microbiota relative to host genomes, opens the possibility of influencing gut health and athletic performance via dietary microbiota manipulation (Scheiman et al., 2019; Lundberg et al., 2021). Altogether, it seems likely that there is a substantial amount of untapped potential concerning the gut microbiome-mucin interactions in athletes and how external factors, such as the consumption of natural or synthetic glycans, may influence this symbiosis.

Therefore, this review aims to give insights into the potential avenues for exercise physiology and gut mucosal health, building on the surge of recent primary research to debate different aspects of the complex bidirectional interaction between gut mucin glycans and microorganisms. Firstly, we will focus on gut mucin composition, function, synthesis, and their synergy with gut bacterial communities, followed by some characteristics of the mucus layer that are of great interest in physiological and pathophysiological contexts for endurance athletes. Secondly, we will propose plausible dietary glycan supplementation that could modify the microbiome-glycans on mucins interconnection and improve, prevent, or maintain healthy gut mucus in athletes.

## Materials and methods

### Eligibility criteria and literature search strategy

A systematic and comprehensive search of electronic databases, including Medline database (https://pubmed.ncbi.nlm.nih.gov/), Scopus, ClinicalTrials.gov, Science Direct, Springer Link, Google Scholar, and EMBASE was done from January 2023 to October 2023. The search process was completed using the keywords: “glycans”, “glycome”, “mucin intestinal tract”, “microbiota”, and “athletes”. The search was not restricted to the type of study (e.g., case-control, prospective cohort studies, randomized control trial, before-and-after study, cross-over randomized control trial), type of sport, sample size, age, gender, ethnicity, geographic localization, or species. However, editorials, systematic and literature reviews, and letters to the editors were excluded. Experiments including individuals with or at risk of health problems and disabilities (type 2 diabetes, overweight, obesity, mental health disorders) were excluded. The studies that focused on specific medical conditions, treatments, or demographics were not included in our analysis. However, we did not exclude studies on colitis and inflammatory bowel diseases since exercise-induced gastrointestinal distress has some similarities with these conditions, such as increased intestinal permeability. We only considered peer-reviewed and original research studies published from 2000 forward and in English only.

### Data extraction

Complete copies of citations coded as potentially relevant were obtained, and those meeting the inclusion criteria were read in detail and data extracted. The authors pulled information about the species, study aims, population, sample size, dietary records, experimental design and duration of follow-up, individual characteristics, changes in the microbiota composition, mucin characterization, and association or not with gastrointestinal damage. The primary outcome was the microbiota profile and mucin composition or structure changes. If eligibility could be determined, the entire article was retrieved. The extracted data included the year the paper was published, the species, the sample size, protocol, and outcomes. All papers were exported to the reference database Mendeley.

### Data synthesis

A search conducted in January 2023 resulted in the following list of crucial term combinations (gut microbiota, mucins, gut permeability = 107; mucin glycome and gut microbiota = 14; mucin, exercise, and gut microbiota = 2; mucin, dietary glycans, and gut microbiota = 134. After the removal of duplicates, 81 article titles and abstracts were screened. During the full-text screening, 14 were excluded for not meeting the inclusion criteria or because one or more of the criteria for exclusion were met. Finally, 67 articles met all criteria for inclusion in the current review. Among them, 24 were related to the gut microbiota, mucins, and gut permeability, 13 were associated with glycome and microbiota, 2 with mucin, exercise, and gut microbiota, and 28 with mucin, dietary glycans, and gut microbiota.

Data collection periods spanned from 2000 to 2023, providing data from human and animal models like mice and horses.

## Results and discussion

### The gut mucin glycans composition and their primary functional roles in the gut

The organization of the mucus along the gastrointestinal tract has only been well characterized over the last decade (Pelaseyed et al., 2014). We now know two types of mucus organization in the gastrointestinal tract (Pelaseyed et al., 2014). The glandular stomach and colon have a two-layered system with inner and outer mucus layers, whereas the small intestine has only one layer (Pelaseyed et al., 2014). The mucus organization in the large intestine matches the fact that the human colon harbors more than a kilogram of bacteria. In the large intestine, the outer mucus layer is less dense and voluminous, not attached (also called loose mucus), and provides a natural habitat and carbon and nitrogen source for the commensal bacteria adapted to low oxygen levels (Bell et al., 2019; Bergstrom et al., 2020). Thus, the outer mucus is a central ecological niche for the microbiota, acting as the primary interface between gut cells and commensal microorganisms (Donaldson et al., 2015).

Conversely, the stratified inner mucus layer is dense and devoid of the microbiota (Johansson et al., 2015) and functions as a protective barrier, minimizing microbial translocation and preventing excessive immune activation (Schroeder et al., 2018). Intestinal mucus also provides dynamic removal of bacteria and limits their access to the epithelium because both mucus layers turn over in hours (Hansson, 2020). Peristalsis in the intestine also helps (Hansson, 2020). Coupled with mucus renewal, the intestinal epithelium surface, including goblet cells, is continuously renewed from the stem cells at the crypt base to ensure epithelial homeostasis and regeneration and has an average cell turnover of 3–7 days (Barker, 2014).

The mucus is mostly water (usually >98%) and mucins, followed by salts, lipids, non-mucin proteins, and immunological factors, varying concentrations depending on gut condition and location. Mucins are a large, complex family of heavily *O*-glycosylated proteins (Bansil and Turner, 2018; Nason et al., 2021). There are two classes of mucins: those that remain tethered to cell membranes and mucins that are secreted, usually by the goblet cells of the small intestine and colon or the surface mucous cells of the stomach (Cone, 2009). The gastrointestinal tract’s main secreted and anchored mucin is mucin 2 (MUC2) (Carvalho et al., 2012). The membrane-anchored MUC2 forms the glycocalyx (Hansson, 2020). The canonical role of MUC2 is to lubricate, hydrate, and protect epithelial surfaces against the outside environment (Goto et al., 2016). Additionally, because gut cell surfaces are a significant site of MUC2 expression, their roles include mediating cell-cell and pathogen-cell interactions. Thus, MUC2 is broadly involved in immune recognition and trapping, immune cell activation or suppression (Schnaar, 2015; Reily et al., 2019; Qin and Mahal, 2021; Brazil and Parkos, 2022), antimicrobial functions (Bergstrom and Xia, 2022) and interaction with microbes (Paone and Cani, 2020).

Most of the functions of MUC2 are governed by the glycosylation patterns of the *O*-linked glycans. *O-*glycans typically make up more than 80% of the mass of mucins (Hansson, 2020). Generally, mucins have a central protein core rich in Ser, Pro, or Thr-repetitive and non-repetitive sequences (Ridley and Thornton, 2018; Hansson, 2019) decorated with many *O*-glycan chains. These *O*-glycans are primarily built from five monosaccharide components: galactose, *N*-acetylglucosamine (GlcNAc), *N*-acetylgalactosamine (GalNAc), fucose, and sialic acid (Ridley and Thornton, 2018; Hansson, 2019), which are attached to the protein backbone through an oxygen atom. Together, they form a dense carbohydrate layer that covers the protein core (Bergstrom and Xia, 2013). Mucin *O*-linked glycans exert canonical functions upon glycosylation (Qin and Mahal, 2021). Glycosylation expression and composition profile in mucins differ among the different gastrointestinal tract regions and individuals (genetic factors partly determine them (Belzer, 2022)). Notably, the process of *O*-glycosylation leads to remarkable *O*-linked glycan heterogeneity and diversity, with more than 200 distinct forms identified in mucins (Varki, 2007). Each possible glycan form has a potentially unique regulatory capability (Jin et al., 2017). The ensemble of glycan forms found in mucins and their arrangements, particularly within glycoproteins, comprise the glycome (Kunej, 2019). The emerging field of glycomics, which evaluates the structures and function of glycoproteins in a biological system (Kunej, 2019), has started to delineate the emerging and promising role of the mucinome, which is the ensemble of glycoproteins whose mucin domains make them functional (Malaker et al., 2022).

There is now a growing appreciation of the need to study the mucin glycans role in gut health. As such, *MUC2*-deficient mice lack a standard intestinal mucus layer and are more susceptible to intestinal inflammation and infection (Van der Sluis et al., 2006). In agreement, *MUC2* ^*−/−*^ mice displayed increased epithelial barrier permeability because of mucus defects and intestinal epithelial tight junction impairment and developed spontaneous colitis (Lu et al., 2011). In another example, mice deficient in intestinal mucin *O-*glycans, T-synthase-knockout mice and Cosmc-knockouts, presented loss of the gut outer and inner intestinal mucus layers and increased bacterial–epithelial contact in the distal colon, as well as intestinal permeability (Fu et al., 2011; Kudelka et al., 2016).

### Gastrointestinal symptoms in endurance athletes: gut mucus damage matters

Over the past decades, the increased popularity of endurance events has raised concerns regarding prolonged and intense exercise’s impact on gastrointestinal health, leading to worse performance (Svendsen et al., 2016) and recovery (Gordon et al., 2017). Around 30%–50% of endurance athletes (Jeukendrup et al., 2000; De Oliveira et al., 2014) and 96% of ultramarathon runners experience acute gastrointestinal distress during or after exercise (Hoffman and Fogard, 2011; Urwin et al., 2021). Although most symptoms are mild and do not cause long-term health effects, epigastric pain, heartburn, nausea, vomiting, abdominal pain, bloody diarrhea episodes, and dehydration are typical responses in endurance athletes (Koehler et al., 2021). Yet, increasing evidence shows that these symptoms may indicate more chronic intestinal damage (De Oliveira et al., 2014), which could impact subsequent recovery.

**FIGURE 2:**
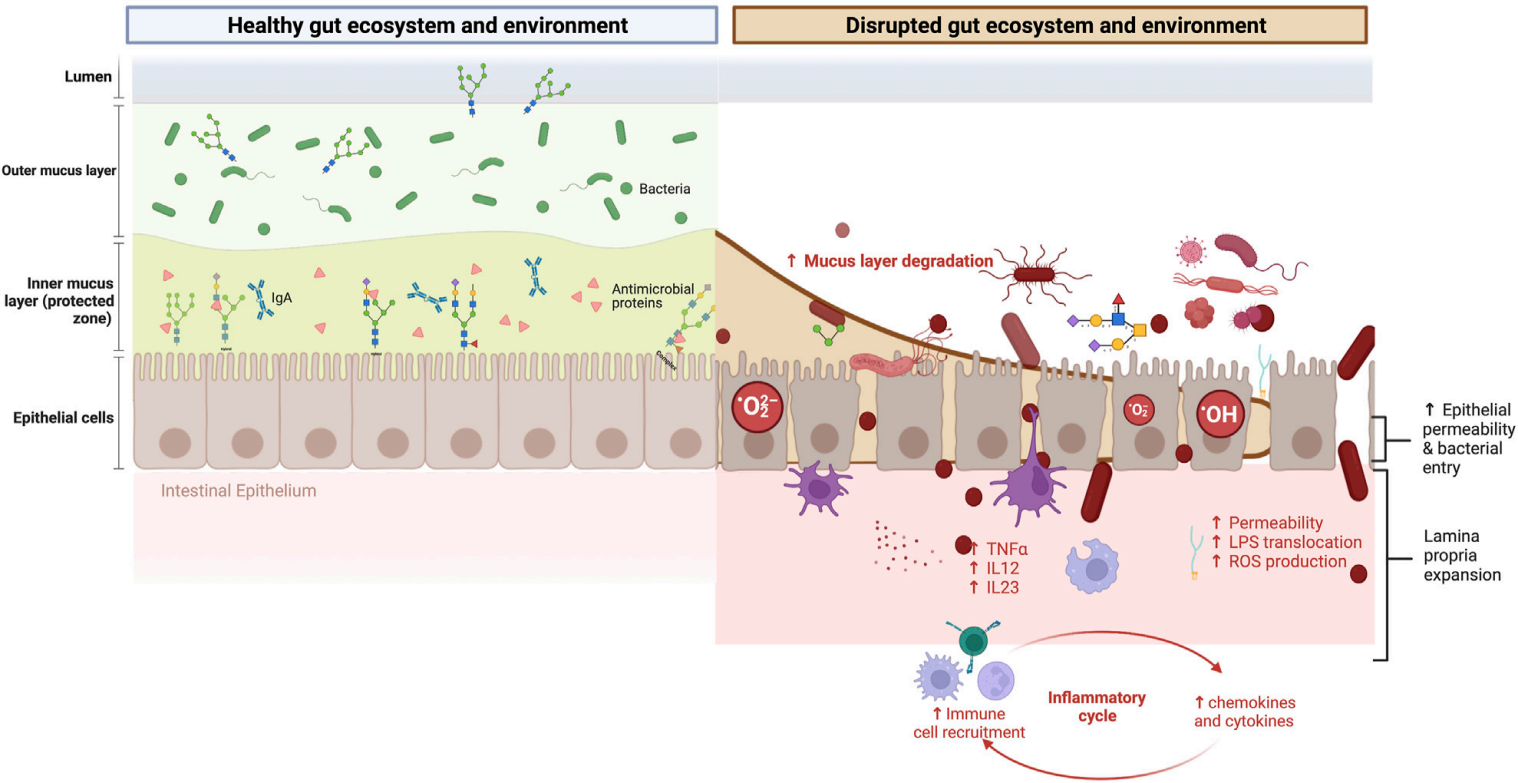
Gastrointestinal symptoms in endurance athletes: mucosal glycome damage matters. Left panel: The mutualistic relationship between the gut mucin glycome, gut microbiota, and derived metabolites under eubiosis. On the one hand, the gut microbiota and their metabolism induce the synthesis of large gel-forming mucins, including encapsulation, glycosylation, changes in fucosylation and sialidation patterns, and thickness. On the other hand, the mucin layer serves as an environmental niche and a food source for the microbiota. The high diversity of gut mucins impacts the gut microbiota composition, diversity, and stability but also influences the immune and mitochondrial function. Right panel: The mutualistic relationship between the gut mucin glycome and the microbiota under a disrupted gut ecosystem and environment. Vigorous endurance training increases sympathetic nervous system activity and redistributes blood flow from the splanchnic organs to the working skeletal muscles and peripheral circulation. This results in gut hypoxia and hypoperfusion, reduced intestinal motility and absorption capacity, damage of the specialized antimicrobial protein-secreting cells (Paneth cells), the mucus-producing goblet cells, and increased loosening of tight junction protein structure and MUC2 destruction. The deterioration of the gastrointestinal mucosal barrier enables the translocation of bacteria and lipopolysaccharides (LPS) outside the gastrointestinal tract, triggering immune and inflammatory responses, often resulting in increased intestinal permeability and, eventually, endotoxemia. Moreover, it induces oxidative stress via mitochondrial ROS production. Changes in the intestinal barrier integrity involve changes in the abundance, expression, and glycosylation of mucins, and thus immune dysregulation, dysbiosis, and risk of disease onset. This figure has been created with BioRender.com.

Until now, the negative symptoms linked to strenuous exercise were believed to be primarily due to gut blood hypoperfusion (Jeukendrup et al., 2000). Vigorous endurance training (≥60 min and ≥70% of maximum work capacity) increases sympathetic nervous system activity. It redistributes blood flow from the splanchnic organs to the working skeletal muscles and peripheral circulation (Mach and Fuster-Botella, 2017). This results in gut hypoxia and hypoperfusion, reduced intestinal motility and absorption capacity, damage of the specialized antimicrobial protein-secreting cells (Paneth cells), the mucus-producing cells (such as goblet cells), and increased loosening of tight junction protein structure (Ribeiro et al., 2021). Once the exercise is finished, the blood flow reperfusion in the gut may also contribute to longer-lasting conditions such as gastritis or ulcers (Waterman and Kapur, 2012). Lastly, mechanical damage to the epithelia caused by general movement, specifically running, is another mechanism linked to gastrointestinal distress (Smith et al., 2021). All these events are not mutually exclusive. The acute deterioration and inflammation of the gastrointestinal mucosal barrier enable the release of immunogenic substances, the translocation of bacteria and bacterial lipopolysaccharides (LPS), and the subsequent toll-like receptor-4 (TLR4) and CD14 activity. Altogether, prompt the release of pro-inflammatory cytokines such as TNFα and IL-1α, leading to chronic low-grade inflammation (Mohr et al., 2020) and intestinal permeability (Stuempfle and Hoffman, 2015; Ruiz-Iglesias et al., 2020; Koehler et al., 2021). The immune response, however, depends on the microorganisms from which LPS originates and the LPS structure (Mohr et al., 2022). Moreover, endurance exercise induces oxidative stress within intestinal epithelial cells via mitochondrial ROS production (Pagesy et al., 2022), as well as changes in the oxygen concentration and osmolarity of the gut lumen (Jeukendrup et al., 2000) (Figure 2). Lastly, emotional stress experienced by many endurance athletes can activate the sympathetic nervous system and hypothalamic-pituitary-adrenal axis and further contribute to gut dysregulation (Silva et al., 2014; Clark and Mach, 2016; Koehler et al., 2021).

Beyond gut disturbances, a common problem affecting elite athletes is the increased incidence of upper respiratory tract infections (O’Brien et al., 2022). The immunosuppressive effect of exercise at high intensity, the chronic systemic inflammation, partly caused by increased gut permeability, and the excessive strain on the respiratory system (relative to its capacity) leads to exercise-induced hypoxemia (Durand and Raberin, 2021) and increased risk of infections (Nieman, 2003; O’Brien et al., 2022). Therefore, gastrointestinal damage may be associated with respiratory comorbidities in top-level endurance athletes due to exertion in extreme conditions and the bidirectional inter-organ communication of the gut-lung axis (Budden et al., 2017; Dang and Marsland, 2019; Mach et al., 2021a). Future investigation should elucidate how the gut-lung axis affects athletic performance and health and how to mitigate exercise-induced gut permeability.

### The gut mucin glycome-microbiota interactions: Two-way traffic

A symbiosis exists between gut mucins glycans (secreted and tethered to cells) and the microbiome.

On the one hand, intestinal mucus is highly efficient at handling a high number of microorganisms (>2,000 different bacterial species), and for this, glycans on mucins are of crucial importance (Hansson, 2020). Mucus-associated microorganisms are found in the outer mucus layer of the colon, where they swim, settle, and communicate with each other (Werlang et al., 2019). These adapted mucosal taxa encode CAZymes to digest most of the repertoire of dietary glycans that arrive in the large intestine (Kaoutari et al., 2013). Dietary glycans come in many forms, from long polysaccharide chains that humans cannot digest (e.g., cellulose, pectins, resistant starch) to oligosaccharide chains attached to proteins and lipids, and mono and disaccharides, such as glucose, lactose, or sialic acids (Koropatkin et al., 2012). Fruits, vegetables, and cereals provide carbohydrates readily digested by human intestinal enzymes and complex dietary glycans, which are resistant to digestion and absorption in the small intestine (Kaoutari et al., 2013). Dietary fibers are one of the most heavily studied groups of dietary glycans. Fiber is a broad term encompassing polysaccharides, oligosaccharides, and resistant starches, namely, inulin, dextrin, pectin, cellulose, resistant starch, arabinoxylans, and chitin (Krautkramer et al., 2020). These complex dietary carbohydrates (in soluble and insoluble forms) evade breakdown by a limited repertoire of host enzymes in the small intestine and pass to the distal gut, which serves as a substrate for many microorganisms (Krautkramer et al., 2020).

Gram-negative Bacteroidetes, Gram-positive Firmicutes, Actinobacteria, and Verrucomicrobia bacterial species encode thousands of CAZymes and associated transport systems that uptake, depolymerize, and ferment dietary glycans into CO_2_, H_2_, and SCFA (Kaoutari et al., 2013; Tailford et al., 2015; Belzer et al., 2017; Plovier et al., 2017; Sicard et al., 2017; Crouch et al., 2020; Taleb et al., 2022).

Interestingly, some taxa can forage host mucin glycans, which provide an additional or alternative energy source for the distal microbes and favors their replication and colonization (Bergstrom and Xia, 2013; Kaoutari et al., 2013; Ndeh and Gilbert, 2018). This ability to adaptively feed on host mucin glycans provides access to a more stable nutrient reservoir, compared with dietary fiber intake composition and abundance, which vary daily and from person to person (Kaoutari et al., 2013). Genes involved in the cleavage of mucin glycans in the gut are found in many bacteria, e.g., in 86% of the 397 genomes of gut microbes analyzed, including taxa from Lachnospiraceae, Enterobacteriaceae, *Bacteroides*, *Eubacterium rectale, Faecalibacterium prausnitzii*, *Eubacterium cylindroides*, *Clostridium histolyticum*, *Clostridium lituseburense,* and *Akkermansia muciniphila* (Ravcheev and Thiele, 2017). Once bacterial glycosidases have started to act on the mucin glycans, the host-specific glycan epitopes will disappear. The bacteria will continue fermenting glycans, and finally, the protein backbone will be exposed and degraded, contributing to complete mucus degradation. Only small amounts of mucin appear in the feces, showing that the commensal bacteria efficiently utilize host mucins (Hansson, 2020). It is also possible that many uncharacterized bacteria, archaea, or other microorganisms can grow on host glycan via cross-feeding activities dependent on other glycan utilizers. In any case, microbial forage of the outer mucus gel layer is considered a normal part of mucin turnover and regeneration (Coker et al., 2021). At the species level, *A. muciniphila* is the best-known taxa for its ability to degrade and utilize mucins, and it is thereby considered a mucin-degrading specialist, playing a significant role in maintaining gut health. This bacterial species encodes the enzymatic processing machinery required for mucin metabolism, including proteases, sulphatases, and glycosyl hydrolases (Trastoy et al., 2020).

On the other hand, the gut microbiota and their metabolites regulate the synthesis of large gel-forming mucins, including encapsulation, glycosylation, changes in fucosylation and sialidation patterns, and thickness (Figure 2). Hence, the microbiota composition and function are important to maintain homeostasis of the gut ecological microenvironment. As shown in gnotobiotic mice, the intestinal epithelium undergoes abnormal development without gut microbiota, leading to defects in mucin secretion and barrier function (Birchenough et al., 2015). Using germ-free mice, Johansson et al., 2015) also confirmed that the gut microbiota is fundamental for forming a proper mucus layer in mice. First, the number of filled goblet cells in conventional mice was higher than germ-free ones. Second, the inner mucus layer in the colon was thicker and, therefore, impenetrable to bacteria compared to germ-free mice. Supporting these insights, human patients with gut dysbiosis (defined as low microbial diversity and an unstable composition over time) presented reduced mRNA expression of secreted and tethered mucins (MUC2, MUC12, MUC13, MUC15, MUC20, MUC21) and immune dysregulation (Lo Conte et al., 2023). Beyond mucin production and abundance, other studies have shown how the gut microbiota regulates not only the production and secretion of MUC2 but also their *O*-glycosylation (Goto et al., 2016; Arike et al., 2017; Bergstrom et al., 2020). As such, Hanson and Hollingsworth (2016) showed that gut microbes in conventionally raised mice express more complex, extended mucin *O*-glycome structures throughout the intestine due to increased levels of many glycosyltransferases. *In vitro* and *in vivo* experiments in dysbiotic diarrheal pigs have shown aberrant mucin *O*-glycosylation patterns, intestinal inflammatory response, and defects in the abundance of the tight junction proteins ZO-1, occludin, and claudin1, all of which are essential for proper intestinal epithelial barrier function (Xia et al., 2022).

While inner mucus and membrane-linked mucins are impenetrable under eubiosis and keep microorganisms at a distance, the microbial metabolites can easily penetrate and influence the formation and composition of membrane-linked mucins. Most SCFAs produced from bacterial mucin fermentation diffuse back through the inner mucus layer and are absorbed in the local epithelial cells (Hansson, 2020). For instance, butyrate produced by intestinal microbiota is oxidized in the colonocytes to generate CO_2_, which can be converted into HCO_3_^−^, an ideal physiological solution for precipitation calcium and raising the pH at the epithelial surface. This, in turn, promotes the stratification of the mucus layer (Fang et al., 2021). Additionally, butyrate upregulates the transcription of genes related to both secreted and membrane-linked mucins. For example, mice receiving rectal enemas of butyrate (100 mM) for seven consecutive days resulted in enhanced synthesis of secreted (*MUC2*) and tethered mucins (*MUC1, MUC3, MUC4*) in the gut (Gaudier et al., 2009). Using *in vitro* models, bioactive SCFA administration (primarily butyrate) promoted *MUC2* and *MUC5AC* gene expression and increased epithelial cell integrity after damage using human intestinal HT29-MTX-E12 cells (Giromini et al., 2022). Both butyrate and propionate epigenetically regulated *MUC2* gene expression in the human goblet cell-like LS174T cells (Burger-van Paassen et al., 2009). Conversely, dysbiosis and the accompanying loss of microbiota-derived metabolites in humans led to a reduced abundance of mucin *O*-glycans and SCFAs (Larsson et al., 2011), confirming the link between microbial-generated metabolites and mucus gut health (Pabst and Slack, 2020).

Besides SCFAs, bacteria and microbial products such as LPS, flagellin, and lipoteichoic acid, among others, can also modify mucin composition and structure. Accordingly, an *in vitro* study in human mucin-secreting goblet cell line HT29-MTX revealed that bacterial LPS increased mucin *MUC5AC, MUC5B*, and cytokine mRNA expression (Smirnova et al., 2003). These keen observations have profound implications for athletes. As previously described, prolonged and excessive exercise stimuli (especially at around 70%–80% of the maximum oxygen consumption) produce splanchnic hypoperfusion and subsequent ischemia that can damage the gut epithelium (Paneth cells, goblet cells, and the tight junction proteins), impacting the intestinal mucosa’s integrity and increasing its permeability to external agents such as LPS and bacteria (Stuempfle and Hoffman, 2015; Ruiz-Iglesias et al., 2020; Koehler et al., 2021). By binding to extracellular TLR4 on many cell types, LPS elicits strong inflammatory and immune responses that may harm the athlete’s gastrointestinal tract (Mohr et al., 2020). Additionally, dehydration and hyperthermia (∼40°), two exercise-related factors that disturb the tight junctions and increase intestinal permeability in athletes (Walter et al., 2021), can enhance the pro-inflammatory immune responses. Changes in gut permeability, characterized by increased plasma lactulose: rhamnose concentration ratio, occurred in adult men performing exercise under hot conditions compared to equivalent passive hyperthermia (Walter et al., 2021).

In this regard, the gut microbiota, the microbial metabolites, mucins, epithelium cells, and tight junctions are likely interdependent during exercise, so the loss of one diminishes the other (Capaldo et al., 2017). Taking advantage of human intestinal enteroids, Pearce et al. (2020) demonstrated that the infusion of microbial-derived SCFA affected the overall gut barrier function, including increased expression of mucin genes, goblet cell markers, common mucus constituents, and antimicrobial peptides (Pearce et al., 2020). Butyrate administration in Caco-2 cell monolayers restored occludin and F-actin delocalization and enhanced tight junction protein expression (Peng et al., 2009). Similar responses were seen in pigs that received gastric infusions of SCFAs (Diao et al., 2019). SCFAs directly affect the transcription of tight junction proteins like occludin and claudin-1 (Diao et al., 2019). Taken together, the increased permeability and compromised barrier function in athletes are most likely due to a combination of ischemia and reperfusion injury of epithelial cells and tight junctions, defects in mucin composition, and modifications of the residing microbiota ecosystem and their metabolites (Keirns et al., 2020; Kudelka et al., 2020).

Despite these findings, mucosal health is multifaceted. Factors such as pH, ionic conditions, water content, gut motility, food consistency, stool passages, mitochondrial metabolic deregulation in the intestinal cells, and stress hormones also regulate host mucus layer glycan expression and structure, increasing the diversification of mucin patterns (Hiippala et al., 2018). For instance, mitochondrial damage and metabolic deficiency in gut epithelial cells have been linked to intestinal permeability in *MUC2* knockout mice (Borisova et al., 2020). Chemically induced mitochondrial uncoupling in untreated C57Bl/6 mice induced the intestinal barrier disruption *in vivo* and caused loss of F-actin and claudin-3 disassembly similar to that found in *MUC2* ^*−/−*^ mice. Using an oxidative Caco-2 model, cell mitochondrial ATP depletion itself caused immediate effects on the barrier integrity via F-actin disassembly and disintegration of the tight junction protein complexes (Janssen Duijghuijsen et al., 2017). Thus, mitochondria from epithelium cells play an essential role in maintaining an intestinal barrier with high integrity. Other work has suggested that repeated bouts of stress decrease MUC2 synthesis and the number of goblet cells via notch signaling suppression in rats (Pfeiffer et al., 2001). Silva and colleagues revealed that stress strongly affects the *O*-glycosylation of mucins, resulting in the flattening and loss of cohesive properties of the mucus layer (Silva et al., 2014). However, not all studies have shown universal effects. Jakobsson et al. (2015) reported that a thinner mucus layer is still functional, showing the power of gut mucins to protect against aggressive communities in these extreme conditions (Bergstrom and Xia, 2022).

Overall, the gut mucin-microbiota interplay represents an exciting area of research with the potential to improve our understanding of gut health and intestinal permeability in normal physiological conditions, such as endurance exercise, and in many disease-related situations. Further research is needed to fully elucidate the mechanisms underlying the interactions between the gut mucin glycans and the gut microbiota and to develop strategies for modulating the gut mucinome-microbiota relation for therapeutic purposes.

### The gut mucin glycome-microbiota relationship depends on dietary glycans

The two-way traffic between gut mucin glycans and microbiota is more complex than previously thought. The interaction between host mucin glycans and microbiota also depends on the dietary glycans (Koropatkin et al., 2012; David et al., 2014). Different dietary glycans can serve as specific substrates for other microbial taxa, influencing their growth and abundance in the gut.

The interactions between dietary and host glycans and the microbiome have essential gut health and disease prevention implications. For example, a diet high in refined carbohydrates and low in fiber decreases the gut microbiota diversity and modifies bacterial genomic regulation. Thus, the intestinal microbial ecosystem equilibrium shifts toward an ecosystem with higher mucin-degrading abilities, increasing the likelihood of colonic outer mucus layer degradation and intestinal inflammation (Egan et al., 2014). Several studies have shown that Western diets and simple and easily digestible sugars, such as glucose, are often associated with adverse effects on intestinal health and microbiome (Pelaseyed et al., 2014; Coker et al., 2021). A study by Desai et al. (2016) illustrated how dietary fiber deficiencies could promote specific gut bacterial populations and their enzymes and cellular metabolism to shift towards host mucin glycans as an energy source, leading to erosion of the colonic mucus layer. For example, *Bacteroides thetaiotaomicron* can degrade host mucus glycans when polysaccharides are absent from the diet (Sonnenburg et al., 2005). In line with these findings, lack of dietary fiber induced defects of the inner colonic mucus layer, which included a strongly reduced growth rate and higher mucus layer penetrability after changing from a chow diet to Western-style diets in mice (Bishara et al., 2013). In support of this notion, Schroeder et al. (2018) observed significantly decreased diversity and reduced abundance of Bacteroidetes and Actinobacteria without fiber for up to 40 days. Precisely, a long-term low-fiber, high-sugar diet triggered an ecological community forced to forage on host mucin glycans, leading to thinning of the mucus layer (Schroeder et al., 2018). This altered mucus increased the risk factor for infection and low-grade inflammation (Bishara et al., 2013; Coker et al., 2021). Conversely, *Bifidobacterium longum* supplementation in mice fed with diets deficient in fiber restored mucus growth (Schroeder et al., 2018). Seemingly, *A. muciniphila* supplementation has been shown to restore gut mucus layer thickness, modulate immune system response and gut barrier function, among others (Shin et al., 2014; Wu et al., 2017; Zhai et al., 2019). Although *A. muciniphila* is a mucus degrader, its oral supplementation increases the number and differentiation of Paneth cells and goblet cells in the small intestine. Subsequently, it accelerates intestinal epithelial regeneration (Kim et al., 2021). Confirming these findings, exposing human enteroids to cecal contents obtained from mice treated with *A. muciniphila* stimulated the MUC2 protein and *MUC2* mRNA expression, along with mucus thickness, compared to the control (Kim et al., 2021).

More recently, it has been shown that the outer-membrane pili proteins of *A. muciniphila* alone are sufficient to improve intestinal barrier function (Ottman et al., 2017) by increasing the number of goblet cells and reinforcing the intestinal barrier (Plovier et al., 2017). These data demonstrate that, on the one hand, the lack of dietary fiber leads to changes in the gut microbiome-mucin interlinkage, promoting gut mucosal barrier dysfunction. On the other hand, the plasticity of the human gut microbiota in response to dietary changes paves the way to use different preparations of *A. muciniphila* and *B. longum* as therapeutic options to target gut athletes’ health and associated exercise-triggered gastrointestinal disorders.

### The gut microbiome-glycome dialogue in endurance athletes

The mechanisms underlying the relationship between the gut microbiota, mucin glycans, and athletic performance have yet to be understood. Still, the ergogenic effects of the gut microbiota involve factors such as nutrient metabolism, energy balance, immune function, and intestinal health. We believe that a functioning and healthy gut mucus barrier is essential to observe the critical contribution of the gut microbiota to athlete physiology and performance. Thus, the gut mucin glycans-microbiome interplay might be the gateway for researchers aiming to influence gut health in athletes.

**FIGURE 3:**
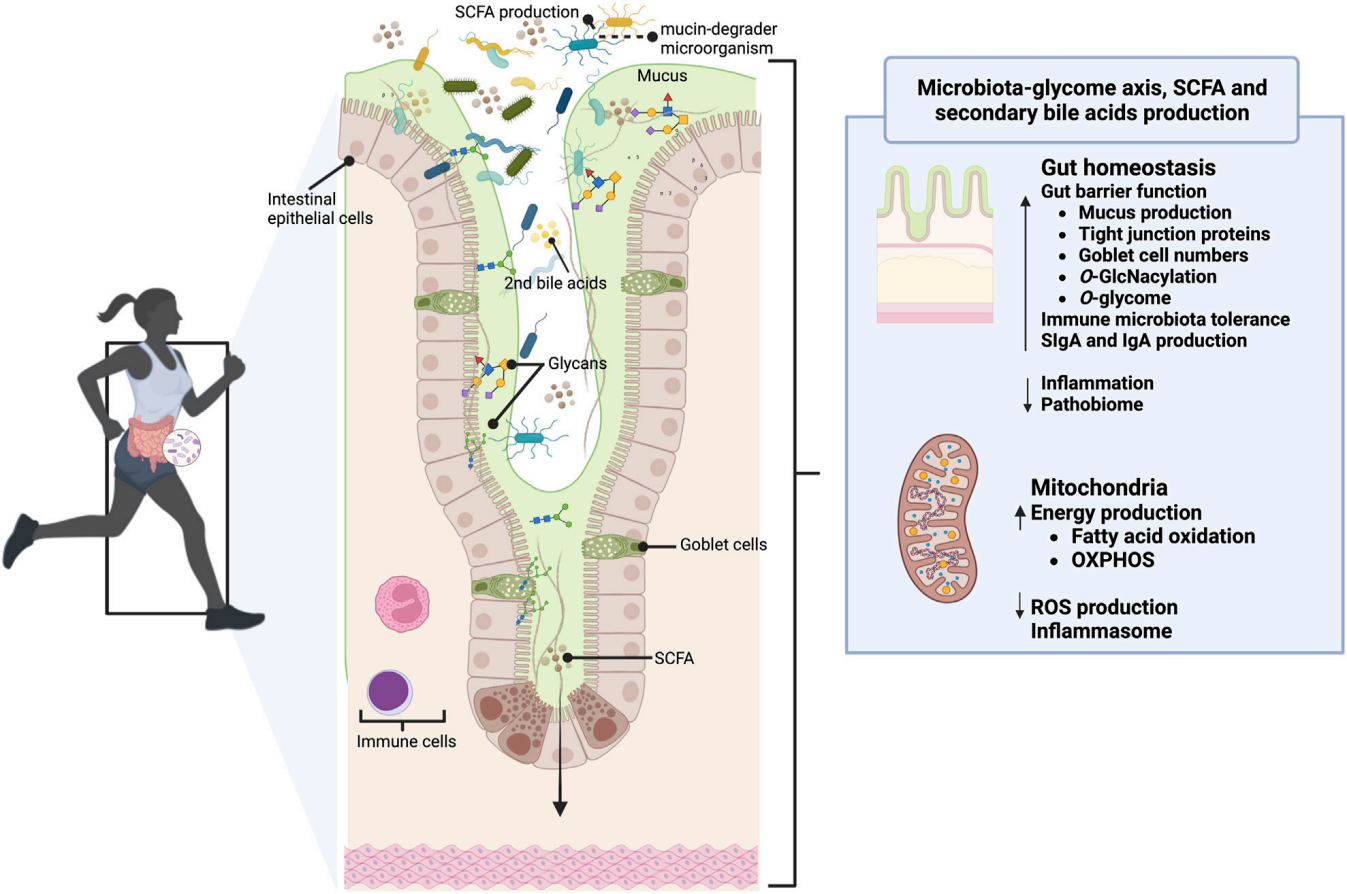
The gut glycome-microbiota interaction in the gut of athletes. In healthy athletes, mucins (mainly MUC2) clear, contain, feed, prevent the proteolytic activity of bacteria and digestive enzymes, and continuously replenish the gut microbiome. The mucinome might modulate the gut microbiome composition and function (*e.g.,* SCFA and secondary bile acids production), and it may be the gateway for researchers aiming to influence gut health. Moreover, its *O-*glycosylation and the might control the nutrient and stress sensing during exercise and the mitochondrial function, illustrating how regulating the glycome-microbiota interplay can be a decisive factor in improving exercise performance. Reciprocally, the athlete’s gut microbiome possesses a functional capacity primed for mucosa repair and a more remarkable ability to harness energy from mucins. This figure has been created with BioRender.com.

In the setting of gut microorganisms and host-gut mucins crosstalk during exercise, preliminary quality associative studies have reported a symbiotic and coevolved relationship between the microbiome-encoded enzymatic machinery capable of digesting host mucins and the exercise performance. For example, a recent study by Morita et al. (2023) shows that *B. uniformis*, a dominant component of the gut microbiota with an expanded glycolytic capability to utilize dietary and endogenous glycans, correlated with improved endurance exercise performance in mice and humans. Similar results were reported using Arabian horses, an outstanding sport model given its well-adapted athletic physiological abilities and the capacity to compete at distances of up to 160 km in a single day, an effort comparable to a human marathon or ultra-marathon runners (Capomaccio et al., 2013). Using this horse endurance model, our team revealed that horses who had a higher cardiovascular capacity harbored a higher intestinal abundance of Verrumicrobia, including the *A. muciniphila* and Actinobacteria taxa, and enrichment in CAZymes able to cleave mucin glycans (Mach et al., 2022). Associated with this glycan enzymatic machinery, we also observed an enrichment of KEGG orthologous groups (KOs) related to glycan biosynthesis and metabolism (K09953, K18770, K12309, K12551, K01137, K03276, K12985, K14459) in horses with the highest cardiovascular capacity. These nuances illustrate the possibility that the makeup of the gut microbiome and its ability to impact host mucin synthesis may determine gut health during exercise. In line with this, Barton et al. (2018) found activation of microbiome pathways involved in glycan biosynthesis in professional rugby athletes compared with more sedentary subjects. The mucin-degrading commensal *A. muciniphila*, which regulates intestinal inflammation and barrier function, was more abundant in athletes’ gut than in sedentary subjects (Clarke et al., 2014; Petersen et al., 2017). Similarly, compared with nonathletes, athletes harbor higher abundances of *Bifidobacterium* (O’Donovan et al., 2020), another known mucin degrader (Milani et al., 2015), and an enhancer of mucus layer growth (Bishara et al., 2013). Despite that, the causal relationship between gut microbiota and exercise remains unknown due to the differences in diet and environment when comparing athletes with non-athletes. Taken all together, Barton et al. (2018) speculated that the athlete’s gut microbiome and their metabolites possess a functional capacity primed for mucosa repair (Figure 3) beyond other physiological strands, e.g., enhance mucus production and increase mucin synthesis, modulation of the immune response modulation, gut-brain axis, hydration, and redox balance.

### Administration of synthetic or natural complex glycans in endurance athletes to improve gut mucin glycome-microbiota interplay

To achieve optimal performance, athletes must fuel, train, and utilize their entire superorganisms (holobiont) (Hughes and Holscher, 2021). Up to now, many endurance athletes are encouraged to consume high amounts of simple carbohydrates, moderate amounts of protein, and low amounts of fiber to avoid potential digestive issues such as gas and distension that high-fiber diets can sometimes cause (Clark and Mach, 2016).

**FIGURE 4:**
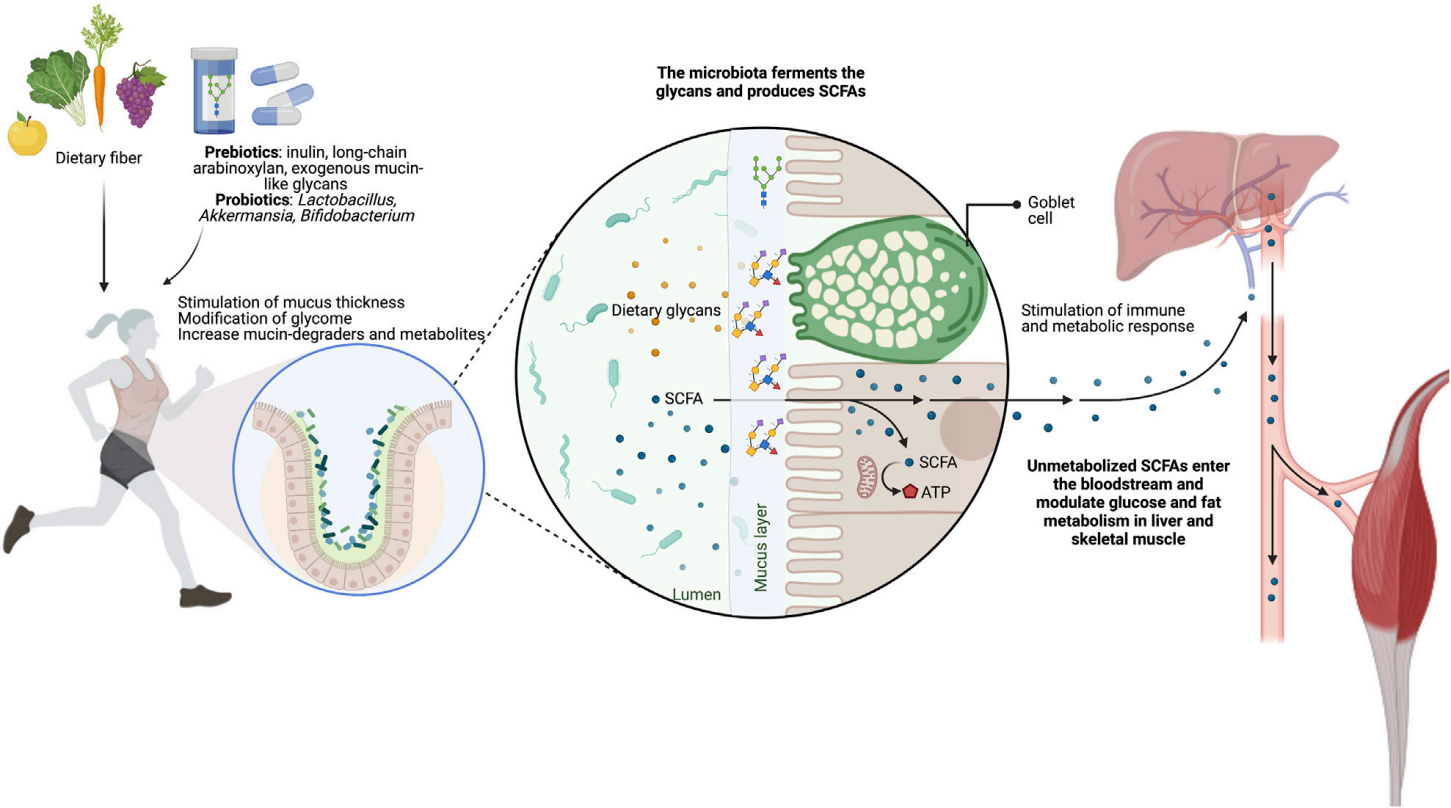
Targeting the gut glycome-microbiota interaction throughout supplementation of glycans. The administration of glycans, such as inulin, long-chain arabinoxylans, and exogenous mucin-like glycans, is an effective strategy to improve or maintain athletes’ intestinal barrier health and function. These nutritional strategies increase the mucus thickness and the SCFA-producing bacteria, both essential for gut homeostasis and health.

Since dietary fiber deficiencies can alter the careful balance between the host mucosal layer’s health and function and the gut microbiome, special attention is needed for the nutritional programs of endurance athletes. Thus, we highlight the need to include complex glycans in athletes’ diets to maintain and improve the gut mucin glycome-microbiota interactions (Figure 4; Table 1). This will complement the benefits of many probiotics and postbiotics tested to improve intestinal barrier integrity potentially, exercise adaptation, and athlete performance (extensively reviewed elsewhere (Jäger et al., 2019; Hughes and Holscher, 2021; Sales and Reimer, 2023)).

**TABLE 1:** Some examples of nutritional strategies that regulate gut mucosal and immune health.

Glycan	Experimental model	Results	References
Dietary fiber	Gnotobiotic mouse model colonized with 14 human gut microbiota species	Bacterial glycan metabolism switched from fiber degradation to mucus layer glycan foraging when rats were fed a fiber-free diet. The gut microbiota used host-secreted mucus glycoproteins as a nutrient source, leading to the erosion of the colonic mucus barrier and increased susceptibility to *Citrobacter rodentium* infection	Desai et al. (2016)
Dietary fiber	Mice fed a fiber-depleted, Western-style diet for 10–40 days	Mice had significantly decreased microbial diversity, reduced abundance of Bacteroidetes and Actinobacteria, and reduced colonic mucus thickness	Schroeder et al. (2018)
Dietary fiber	Gnotobiotic and human-microbiota colonized mice fed with a diet free of microbiota accessible carbohydrates (MACs)	The MACs diet resulted in thinner mucus in the distal colon, increased proximity of microbes to the epithelium, and increased expression of the inflammatory marker REG3β. Increased *Helicobacter pylori* invasion was also observed in mouse stomach gland cells	Earle et al. (2015)
Dietary fiber	Humanized gnotobiotic Fut2^−^ and Fut2^+^ mice were colonized with the *Bacteroides* thetaiotaomicron and were fed a glucose-rich plant polysaccharide-deficient diet for 4 weeks	The B. thetaiotaomicron fucose catabolic pathway gene expression was dependent upon the host diet and polysaccharide content in Fut2−mice. The changes in B. thetaiotaomicron gene expression were only evident in mice fed a plant polysaccharide-deficient diet because B. thetaiotaomicron relies on host mucus consumption	Kashyap et al. (2013)
Inulin	C57BL/6 male mice were fed a high-fat chow diet or a high-fat chow diet supplemented with either inulin or cellulose for 4 weeks	Inulin protected mice against high-fat diet-induced metabolic syndrome by increasing mucosal IL-22 production, which improved enterocyte proliferation and anti-microbial gene expression compared to mice fed just the high-fat diet who experienced bacterial infiltration in the mucus layer. Inulin induced a fortification of the mucosa, with increased barrier functions	Zou et al. (2018)
Inulin and pectin	Wistar rats were fed a normal diet or supplemented with either inulin or pectin for 30 days	Inulin and pectin both stimulated MUC2 production at similar levels compared to controls	Xie et al. (2020)
Inulin and long-chain arabinoxylans	Axenic rats were inoculated with a human fecal microbiota for 6 weeks and were then fed inulin and/or long-chain arabinoxylans	Inulin and long-chain arabinoxylans stimulated the growth of butyrate-producing bacterial groups (*Roseburia intestinalis, Eubacterium rectale, Anaerostipes caccae*) and bifidobacteria (*B. longum*), which resulted in a shift in mucin degradation to distal regions where mucin-degraders may produce beneficial metabolites (e.g*.,* propionate by *Akkermansia muciniphila*)	van den Abbeele et al. (2011)
Inulin-type fructans	5-week old male germ-free Wistar rats were inoculated with human fecal flora and were fed either a standard diet or a standard diet supplemented with an oligofructose and inulin mixture for 28 days	Inulin-type fructan consumption led to changes in the colonic mucosa, increased mucus production, and shifts in the components of mucin components in goblet cells and the epithelial mucus layer. The increased mucus layer prevented *Salmonella Typhimurium* from translocating into the Peyer’s patches	Kleessen and Blaut (2005)
Mannan oligosacchardies (MOS)	5 x FAD transgenic Alzheimer’s disease mice were fed the prebiotic MOS for 8 weeks (0.12%, w/v in the drinking water)	MOS prevented intestinal barrier damage, increased the relative abundance of *Lactobacillus,* and increased butyrate production	Liu et al. (2021)
Sulfate polysaccharides from algae	6-week-old C57BL/6 mice were randomized into 3 test groups: natural control group (NC), 1% and 2.5% dextran sulfate sodium -fed model group (MD), and 100 mg/kg *Gloiopeltis furcata*/day treated group (SAO) for 7 days, followed by 7 days of regular drinking water	SAO algae increased the proportions of complex long-chain mucin *O*-glycans in the epithelial layer with two terminal *N*- acetylneuraminic acid residues while improving the growth of probiotic bacteria, including *Roseburia* spp. and *Muribaculaceae*	Pan et al. (2022)
*O*-glycan-like human milk oligosaccharides (HMOs)	6-week-old GF mice were bi-colonized with two HMO-utilizing taxa common to the infant gut: *Bacteroides* and *B. infantis*. Mice were fed a microbiota-accessible carbohydrates deficient diet supplemented with HMOs (1% in water for 1 week	Supplementing with *O*-glycan-like HMOs or mucin-type *O*-glycans purified from commercially derived porcine gastric mucin aided in recovering lost microbiota post-antibiotic treatment and increased the relative abundance of *A. muciniphila*, suggesting that mucus is a key component of the symbiosis between host and microbiota	Pruss et al. (2021)
Human milk oligosaccharides (HMOs)	HMO-concentrate from donor breast milk was orally administered to 32 healthy adults for 7 days followed by 21 days of monitoring. Fecal samples were collected for 16S rRNA gene sequencing, shotgun metagenomics, and metabolomics analyses	Consuming the HMO-concentrate caused a dose-dependent increase in *Bifidobacterium,* a known HMO consumer, as well as a significant increase in the SCFAs butyrate and acetate suggesting HMOs exert prebiotic functions in the gut. Once HMO consumption stopped, *Bacteroides* increased through day 28, which was associated with elevated TGFβ and IL-10	Jacobs et al. (2023)
Human milk oligosaccharides (HMOs)	Irritable bowel syndrome patients (n = 61) were randomized to consume either placebo, 5 g, or 10 g doses of a 4:1 mix of 2′-O-fucosyllactose (2′FL) and lacto-N-neotetraose (LNnT) for daily 4 weeks	Participants who received 10 g of 2′FL and LNnT experienced an increased in *Bifidobacterium* adolescentis and Bifidobacterium longum as well as changes in fecal and plasma metabolites which were associated with Bifidobacterium spp. Faecalibacterium relative abundance was also increased compared to the placebo group	Iribarren et al. (2021)
Human milk oligosaccharides (HMOs)	Glycomic and genomic fecal analysis was performed to study the fecal microbiome and HMO content of two healthy breastfeeding infants	After the first 2 weeks of life, the fecal HMO content decreased which was associated with an increase in HMO-consuming Bacteroidaceae and Bifidobacteriaceae bacteria. HMO structure analysis revealed that one function of HMOs is to selectively enrich saccharolytic bacteria and that bacterial HMO consumption is highly structure-specific	De Leoz et al. (2015)
Fructo-oligosaccharides (FOS)	Ten patients with active ileocolonic Crohn’s disease consumed 15 g of FOS for 3 weeks	There was a significant decrease in disease score (Harvey Bradshaw index from 9.8 to 6.9). Fecal bifidobacteria concentrations increased significantly. Mucosal IL-10 positive dendritic cells as well as TLR2 and TLR4 expression increased	Lindsay (2006)
Fructo-oligosaccharides (FOS)	High-fat diet-induced C57BL/6 J mice were supplemented with 10% FOS in their water for 6 weeks	FOS increased the expression of numerous genes involved in mucus production, glycosylation and secretion, the expression of both secreted and transmembrane mucins, and the differentiation and number of goblet cells. The relative and absolute abundance of bacteria positively associated with the mucus layer such as *Akkermansia*, were increased	Paone et al. (2022)
Fructo-oligosaccharides (FOS) and galactooligosaccharides (GOS)	Mice were fed a high-fat diet and gavaged with FOS and GOS for 16 weeks	FOS/GOS supplementation improved microbiota dysbiosis (measured as enhanced Firmicutes: Bacteriodetes ratio and reduced biodiversity), tight junction protein claudin1, claudin15, ZO-1, and JAM-A expression was downregulated, and reversed inflammatory cytokines (including TNFα, IL6, and IL17)	Zhang et al. (2021)
Mushroom polysaccharides (Poria cocos)	C57BL/6J ob/ob mice were fed *Poria cocos* by gavage (1.0 g·kg−1, 0.5 g·kg−1) daily for 4 weeks	*Poria cocos* improved the gut mucosal integrity and activated the intestinal PPAR-γ pathway. Increased abundances of butyrate-producing bacteria, mainly *Lachnospiracea* and *Clostridium* were also observed	Sun et al. (2019)
Mushroom polysaccharides (*Auricularia auricular-judae* (*Bull.*)	BALB/C DSS-induced colitis mice were fed either 20 or 40 mg/kg of the mushroom polysaccharide Aap or control feed for 21 days	Glycan consumption improved colon damage and mucosal inflammation and prevented intestinal barrier damage by reducing the D-lactic acid and diamine oxidase levels in plasma	Zhao et al. (2020)
Insoluble yeast β-glucan	High-fat diet-fed rats were supplemented with insoluble yeast β-glucan for 24 weeks	Rats showed less intestinal inflammation and oxidative stress. High-fat diet-induced dysbiosis, lower levels of SCFAs and high levels of LPS were restored. Intestinal barrier function improved by upregulating tight junction proteins and MUC2	Mo et al. (2022)

Current examples of dietary glycans already show their potential to resolve mucus-layer defects associated with disorders such as type 2 diabetes, obesity (O’Keefe et al., 2015; Zhao et al., 2018; Baxter et al., 2019), and gastrointestinal pathologies (e.g., Crohn’s disease, ulcerative colitis, and colorectal cancer (Bischoff et al., 2014)) (see Table 1). Glycans with documented health benefits currently marketed as prebiotics include fructooligosaccharides (FOS), galactooligosaccharides (GOS), xylooligosaccharides (XOS), pullulan, lactulose, and inulin (see the review by O’Brien et al. (2022); Hughes and Holscher (2021)). Broadly, 5–10 g/day of FOS and GOS are needed to spur changes in fecal bacteria, SCFA concentrations (West et al., 2012), and gut health (Roberts et al., 2016). Other studies show that inulin, a water-soluble dietary fiber obtained from chicory, and the long-chain arabinoxylans, an important non-starch polysaccharide in cereal grains, increase colonic mucus thickness as well as gut microbial SCFAs production (van den Abbeele et al., 2011; Schroeder et al., 2018). Both inulin and long-chain arabinoxylans shift the microbial production of acetate towards more health-promoting propionate and butyrate-producers (*Roseburia intestinalis, E. rectale, Anaerostipes caccae*) and bifidobacteria (*B. longum*) (van den Abbeele et al., 2011) while increasing the levels of *A. muciniphila*. Notably, fecal transplantation from mice fed a high microbiome-accessible carbohydrate diet and administered a single portion of inulin increased the fecal SCFA content and improved treadmill running time (Okamoto et al., 2019). On top of this, long-chain arabinoxylans shifted mucin degradation to distal regions, where mucin-degraders may produce beneficial metabolites (e.g., butyrate by *A. muciniphila*) so that prebiotics could improve gut health along the length of the intestine. This is of great interest since most digestive troubles in athletes originate in the distal colon. In line with these results, accumulating studies suggest that dietary polyphenols may stimulate the bloom of *A. muciniphila in vivo* (Henning et al., 2017) and induce the production of polyphenol-beneficial metabolites, further contributing to intestinal health (Rodríguez-Daza et al., 2021). However, it is essential to note that building a stable microbial community after administering prebiotics such as inulin or polyphenols could take several days to a week (van den Abbeele et al., 2011).

Apart from prebiotic glycans expressed by microorganisms or derived from plant materials, processed mucins isolated from pigs and cows have also become a field of interest in modulating gut microbiota (Werlang et al., 2019). Pruss et al. (2021) have recently shown that supplementing *O*-glycan-like human milk oligosaccharides (HMOs) or mucin-type *O*-glycans purified from commercially derived porcine gastric mucin modified the functionality of dysbiotic microbiota. After glycan supplementation, these dysbiotic communities were shifted back to a eubiotic (healthy-associated) state (Pruss et al., 2021). They also demonstrated that mice receiving oral porcine mucin glycans suppressed *Clostridium difficile* abundance after antibiotic treatment, delayed the onset of diet-induced obesity, and increased the relative quantity of *A. muciniphila*. Likewise, rats fed the *O*-glycan GlcNAc produced higher levels of the anti-inflammatory *n*-butyrate and IgA-producing cells in colonic lamina propria than Wistar rats fed porcine stomach proteins (Hino et al., 2020). The increased secretory IgA might prevent toxic and pathogenic gut invasion (Raskova Kafkova et al., 2021), a critical phenomenon in endurance athletes.

Ultimately, evidence demonstrating the ergogenic effects of dietary glycans needs to be improved, and more research is required to substantiate the impact of prebiotics on athletic performance using different human populations.

## Conclusion

An increasing number of clinical problems outside and inside the gut have emerged as one of the most critical factors affecting endurance athletes. The gastrointestinal tract is covered by large, highly glycosylated gel-forming mucins, which are canonical for gut lubrication, hydration, protection, and immune response. Recent evidence shows a complex bidirectional interaction between the gut mucins and the gut microbiota, yet research has just scratched the surface in the context of exercise. Several studies have shown that the mucin glycans composition can impact the microbiome’s composition and function. Reciprocally, the microbiome can also affect the composition and function of the gut mucin glycans. Since mucus mucins are essential for maintaining gut homeostasis and health in athletes, the ergogenic effect of this intertwined connection needs to be explored. Moreover, consuming natural or synthetic complex dietary glycans, an uncommon practice in elite athletes, coupled with probiotics such as *A. muciniphila* and *Bifidobacterium,* might change the gut ecosystem, benefiting mucosal health and preserving the integrity and function of the mucus layer. Overall, the mucin glycans-microbiome interconnection represents an exciting area of research with the potential to improve our understanding of athlete gut health and disease and to develop strategies for modulating the glycans-microbiome interplay for therapeutic and preventive purposes in athletes.
